# Supplementary material for: The dorsomedial prefrontal cortex computes task-invariant relative subjective value for self and other
Source: eLife. 2019 Jun 13;8:e44939. doi: 10.7554/eLife.44939 (PMC6565363; doi:10.7554/eLife.44939)
Supplement: Figure 1—source data 1. — An approximation of the Bayes factor was calculated from BIC values for the 2 k model relative to the 1 k model for the intertemporal choice task, as well as for the 1 α model relative to the 2 α model for the risky choice task. Higher values thus indicate a higher probability of the 2 k model for intertemporal choice and the 1 α model for risky choice. Individual parameters for the best fitting model for each participant are also displayed. [file elife-44939-fig1-data1.pdf]

Figure 1–source data 1: Bayes factor approximation for behavioral model fitting

Intertemporal, 2*k* versus 1*k* model			Risk, 1*α* versus 2*α* model		
**Participant**	**Bayes Factor**	**Parameters 2*k* (*k_self*, *k_other*, *β*)**	**Participant**	**Bayes Factor**	**Parameters 1*α* (*α_shared*, *β*)**
IC1	2.15e+07	0.0066, 6.65e-04, 0.9943	RI1	2.25e+09	1.0366, 0.4710
IC2	9.36e+10	0.0176, 4.53e-04, 0.6683	RI2	3.27e+06	1.7897, 0.0409
IC3	63.67	0.0038, 8.68e-04, 0.5742	RI3	2.31e+05	1.2556, 0.4671
IC4	1.07e+11	0.0092, 3.45e-04, 0.9432	RI4	3.60e+04	1.1899, 0.4465
IC5	8.51	0.0020, 6.47e-04, 0.6367	RI5	1.42	0.9893, 0.8758
IC6	3.81	6.75e-04, 6.36e-05, 0.8013	RI6	2.83e+04	1.1869, 0.6007
IC7	1.80e+04	0.0031, 0.0240, 0.4999	RI7	2.24e+03	1.0147, 0.5449
IC8	1.43e+05	0.0059, 7.16e-04, 0.7027	RI8	4.33e+40	1.0851, 0.2628
IC9	7.02e+12	0.0362, 6.55e-04, 0.5535	RI9	3.59e+11	1.2120, 0.3684
IC10	3.78e+05	0.0019, 1.10e-04, 1.1551	RI10	20.64	1.1738, 0.7934
IC11	1.70e+08	0.0020, 0.0152, 0.6607	RI11	520.00	0.9798, 0.8677
IC12	6.86	0.0015, 6.55e-04, 1.0756	RI12	3.35e+10	1.9955, 0.0272
IC13	9.21e+04	0.0070, 0.0441, 0.6590	RI13	5.90e+04	1.0769, 0.4396
IC14	9.67e+14	2.47e-05, 0.0466, 0.5846	RI14	2.92e+05	1.0290, 0.9631
IC15	1.86e+12	6.93e-04, 0.0280, 0.5566	RI15	3.27e+06	1.2540, 0.3702
IC16	8.39e+14	6.43e-04, 0.0239, 1.1254	RI16	7.37e+09	0.9272, 0.3444
IC17	5.24e+17	0.0011, 0.0497, 0.6398	RI17	2.48	1.1920, 0.4780
IC18	2.06e+06	0.0085, 0.0850, 0.5876	RI18	3.27e+10	0.9736, 0.1555
IC19	2.11e+06	0.0036, 0.244, 0.9836	RI19	4.88e+53	1.1686, 0.3869
IC20	5.74e+03	0.0140, 0.0736, 1.0033	RI20	6.22e+14	1.3210, 0.3633
			RI21	2.58e+11	1.2443, 0.3327

Related to Figure 1. An approximation of the Bayes factor was calculated from BIC values for the $2k$ model relative to the $1k$ model for the intertemporal choice task, as well as for the 1α model relative to the 2α model for the risky choice task. Higher values thus indicate a higher probability of the $2k$ model for intertemporal choice and the 1α model for risky choice. Individual parameters for the best fitting model for each participant are also displayed.
