# Supplementary material for: The dorsomedial prefrontal cortex computes task-invariant relative subjective value for self and other
Source: eLife. 2019 Jun 13;8:e44939. doi: 10.7554/eLife.44939 (PMC6565363; doi:10.7554/eLife.44939)
Supplement: Figure 2—source data 1. — Other over Self trials GLM for intertemporal choice thresholded at p<0.05 FWE-corrected, cluster-defining threshold p<0.001. Neurosynth term search thresholded at p<0.01, FDR-corrected. Only clusters with greater than 40 voxels listed for Neurosynth term search. Coordinates were reported in accordance with the Montreal Neurological Institute (MNI) Atlas. [file elife-44939-fig2-data1.pdf]

Figure 2–source data 1: Other over Self trials GLM contrast for the intertemporal choice paradigm and "social" term search on Neurosynth

Analysis	Voxels	Max *z*	Max MNI Coordinates	Area	Side
Other over Self trials GLM, intertemporal choice	587	4.71	-52 36 -6	Frontal pole	L
	463	4.60	-62 -26 -14	Middle temporal gyrus, posterior division	L
	417	4.42	-48 -56 46	Angular gyrus	L
	244	4.55	-8 42 -20	Frontal medial cortex	L
	166	4.29	-4 52 36	Superior frontal gyrus	L
	92	4.23	26 -82 -42	Cerebellum	R
Neurosynth term search for "social"	1667	9.63	2 58 20	Frontal pole	R
	957	7.28	50 -44 10	Middle temporal gyrus	R
	773	7.47	0 26 -24	Subcallosal cortex	N/A
	743	7.77	54 12 -34	Temporal pole	R
	731	7.43	-36 24 -18	Frontal orbital cortex	L
	650	8.36	-58 -60 20	Middle temporal gyrus, temporoccipital part	L
	566	9.26	-2 -56 36	Precuneous cortex	L
	417	7.82	18 -2 -14	Right amygdala	R
	390	8.13	46 26 -12	Frontal orbital cortex	R
	248	7.03	-20 -6 -18	Left amygdala	L
	208	6.68	46 -42 -22	Inferior temporal gyrus, temporoccipital part	R
	88	5.64	-10 30 58	Superior frontal gyrus	L
	52	5.73	4 10 -4	Subcallosal cortex	R

Related to Figure 2. Other over Self trials GLM for intertemporal choice thresholded at $P < 0.05$ FWE-corrected, cluster-defining threshold $P < 0.001$. Neurosynth term search thresholded at $P < 0.01$, FDR-corrected. Only clusters with greater than 40 voxels listed for Neurosynth term search. Coordinates were reported in accordance with the Montreal Neurological Institute (MNI) Atlas.
