# Supplementary material for: The dorsomedial prefrontal cortex computes task-invariant relative subjective value for self and other
Source: eLife. 2019 Jun 13;8:e44939. doi: 10.7554/eLife.44939 (PMC6565363; doi:10.7554/eLife.44939)
Supplement: Figure 3—source data 1. — Whole-brain thresholded at p<0.05 FWE-corrected, cluster-defining threshold p<0.001. Coordinates were reported in accordance with the Montreal Neurological Institute (MNI) Atlas. [file elife-44939-fig3-data1.pdf]

**Figure 3–source data 1: Relative subjective value (RSV) GLM for Self and Other trials in the intertemporal and risky choice paradigms**

| Analysis | Voxels | Max *z* | Max MNI Coordinates | Area | Side |
|---|---|---|---|---|---|
| Negative RSV GLM, intertemporal choice | 1649 | 5.49 | 36 -82 -18 | Lateral Occipital Cortex, inferior division | R |
| | 945 | 5.72 | 44 -44 44 | Supramarginal Gyrus, posterior division | R |
| | 798 | 5.07 | -50 -58 -22 | Inferior Temporal Gyrus, temporooccipital part | L |
| | 619 | 5.45 | -50 -40 44 | Supramarginal Gyrus, anterior division | L |
| | 534 | 4.57 | 8 22 48 | Paracingulate Gyrus | R |
| | 468 | 4.92 | 40 58 -6 | Frontal Pole | R |
| | 385 | 4.48 | 42 32 14 | Inferior Frontal Gyrus, pars triangularis | R |
| | 382 | 5.09 | -44 46 -8 | Frontal Pole | L |
| | 222 | 4.55 | 34 28 -10 | Frontal Orbital Cortex | R |
| | 208 | 5.51 | -42 18 -6 | Frontal Orbital Cortex | L |
| | 159 | 4.51 | -50 6 22 | Precentral Gyrus | L |
| | 137 | 3.90 | 48 12 24 | Inferior Frontal Gyrus, pars opercularis | R |
| | 118 | 4.52 | 34 12 48 | Middle Frontal Gyrus | R |
| Positive RSV GLM, intertemporal choice | 263 | 4.82 | -58 -40 24 | Parietal Operculum Cortex | L |
| | 154 | 4.25 | 0 46 -4 | Paracingulate gyrus | N/A |
| Negative RSV GLM, risk | 9096 | 6.38 | -6 -90 -8 | Lingual Gyrus | L |
| | 2059 | 5.84 | 14 -70 56 | Lateral Occipital Cortex, superior division | R |
| | 1522 | 6.74 | -42 6 38 | Middle Frontal Gyrus | L |
| | 1380 | 6.36 | 0 14 52 | Paracingulate Gyrus | N/A |
| | 993 | 5.96 | 46 8 36 | Precentral Gyrus | R |
| | 539 | 5.89 | 34 2 48 | Middle Frontal Gyrus | R |
| | 442 | 5.32 | -30 24 -4 | Insular Cortex | L |
| | 280 | 5.35 | 34 24 0 | Insular Cortex | R |
| | 132 | 4.28 | 8 -74 -24 | Occipital Fusiform Gyrus | R |
| | 121 | 4.64 | -2 -30 -4 | Brain-Stem | L |
| Positive RSV GLM, risk | 1396 | 5.04 | 58 -24 22 | Parietal Operculum Cortex | R |
| | 1160 | 5.09 | -62 -30 18 | Parietal Operculum Cortex | L |
| | 828 | 4.67 | 0 42 -8 | Paracingulate Gyrus | N/A |
| | 485 | 4.78 | 10 -92 20 | Occipital Pole | R |
| | 414 | 4.42 | -52 -62 20 | Middle Temporal Gyrus, temporooccipital part | L |
| | 371 | 4.52 | 58 -56 4 | Middle Temporal Gyrus, temporooccipital part | R |
| | 358 | 4.63 | -56 6 0 | Precentral Gyrus | L |
| | 338 | 4.26 | 0 2 44 | Cingulate Gyrus, anterior division | N/A |
| | 333 | 4.51 | -26 0 -24 | Parahippocampal Gyrus, anterior division | L |
| | 305 | 4.87 | -22 -46 62 | Superior Parietal Lobule | L |
| | 295 | 4.48 | 24 -48 58 | Superior Parietal Lobule | R |
| | 167 | 4.37 | 48 18 -36 | Temporal Pole | R |
| | 121 | 4.38 | 18 -4 -18 | Right Amygdala | R |
| Other over Self negative RSV GLM, intertemporal choice | 92 | 4.35 | 4 34 38 | Paracingulate Gyrus | R |
| Other over Self negative RSV GLM, risk | 110 | 4.12 | 12 -60 26 | Precuneous Cortex | R |
| | 95 | 4.01 | 4 -32 46 | Cingulate Gyrus, posterior division | R |

**Related to Figure 3**. Whole-brain thresholded at $P < 0.05$ FWE-corrected, cluster-defining threshold $P < 0.001$. Coordinates were reported in accordance with the Montreal Neurological Institute (MNI) Atlas.
