# Supplementary material for: The dorsomedial prefrontal cortex computes task-invariant relative subjective value for self and other
Source: eLife. 2019 Jun 13;8:e44939. doi: 10.7554/eLife.44939 (PMC6565363; doi:10.7554/eLife.44939)
Supplement: Figure 3—source data 2. — Whole-brain thresholded at p<0.05 FWE-corrected, cluster-defining threshold p<0.001. Coordinates were reported in accordance with the Montreal Neurological Institute (MNI) Atlas. [file elife-44939-fig3-data2.pdf]

Figure 3–source data 2: GLM analysis of offline subjective value recapitulating the methods of Nicolle et al., Neuron, 2012 in the intertemporal choice task

Contrast	Voxels	Max *z*	Max MNI Coordinates	Area	Side
Offline subjective value, Self and Other trials	7886	10.1	-4 -92 -16	Occipital Pole	L
	7323	10.2	30 -88 -16	Occipital Fusiform Gyrus	R
	1147	7.44	44 36 10	Frontal Pole	R
	867	5.84	10 10 52	Paracingulate Gyrus	R
	622	5.97	-46 10 26	Inferior Frontal Gyrus	L
	556	4.96	2 -34 -4	Brain-Stem	R
	398	6.16	30 20 0	Insular Cortex	R
	385	6.15	-36 20 0	Insular Cortex	L
	125	5.60	24 -34 -46	Brain-Stem	R
	111	4.86	28 -2 42	Precentral Gyrus	R
Offline subjective value, Self trials only	15172	8.19	-10 -94 -20	Occipital Pole	L
	1335	5.11	46 26 14	Inferior Frontal Gyrus	R
	1034	5.47	4 16 40	Paracingulate Gyrus	R
	737	5.16	-48 22 26	Middle Frontal Gyrus	L
	285	4.49	20 -30 -4	Cingulate Gyrus, posterior division	R
	244	5.09	-30 22 48	Middle Frontal Gyrus	L
	243	5.05	38 22 4	Frontal Operculum Cortex	R
	212	5.76	34 -2 46	Middle Frontal Gyrus	R
	136	5.03	22 -38 -46	Cerebellum	R
	83	4.30	-26 -38 -48	Cerebellum	L
Offline subjective value, Other trials only	4056	7.24	-36 -80 -22	Lateral Occipital Cortex, inferior division	L
	3656	7.65	40 -82 -20	Lateral Occipital Cortex, inferior division	R
	605	5.72	50 32 24	Middle Frontal Gyrus	R
	330	6.08	2 12 46	Paracingulate Gyrus	R
	311	5.04	-38 18 -6	Insular Cortex	L
	279	6.23	36 20 0	Insular Cortex	R
	130	4.60	-18 -30 -6	Parahippocampal Gyrus, posterior division	L
	130	4.95	-44 -44 40	Supramarginal Gyrus, posterior division	L
	116	4.71	-40 -22 42	Postcentral Gyrus	L
	111	5.38	-52 8 30	Precentral Gyrus	L
	104	5.17	52 -38 48	Supramarginal Gyrus, posterior division	R
	100	5.42	18 -68 6	Intracalcarine Cortex	R

Related to Figure 3. Whole-brain thresholded at $P < 0.05$ FWE-corrected, cluster-defining threshold $P < 0.001$. Coordinates were reported in accordance with the Montreal Neurological Institute (MNI) Atlas.
