# Supplementary material for: The dorsomedial prefrontal cortex computes task-invariant relative subjective value for self and other
Source: eLife. 2019 Jun 13;8:e44939. doi: 10.7554/eLife.44939 (PMC6565363; doi:10.7554/eLife.44939)
Supplement: Figure 4—source data 1. — Dorsomedial prefrontal cortex (dmPFC), ventromedial prefrontal cortex (vmPFC), and an intermediate area (imPFC) spherical ROIs were grown to 8 mm based on peak voxels from previous studies for MVPA. Please note that small dmPFC and vmPFC spherical ROIs grown to 5 mm were used to extract GLM effect sizes. Anatomical ROIs for dmPFC and vmPFC were also taken from previous studies. COG stands for center of gravity. Coordinates were reported in accordance with the Montreal Neurological Institute (MNI) Atlas. [file elife-44939-fig4-data1.pdf]

Supplementary Table 4: ROIs generated for multivariate analyses

ROI	Voxels	COG MNI Coordinates	Side
dmPFC, big	257	0 22 38	N/A
dmPFC, small	81	0 22 38	N/A
imPFC	257	0 42 20	N/A
vmPFC, big	257	0 40 -4	N/A
vmPFC, small	81	0 40 -4	N/A
S1	257	48 48 -20	R
dmPFC, anatomical	1539	12.6 30.6 40.4	R
	1387	-13.0 30.6 41.0	L

Related to Figure 4. Dorsomedial prefrontal cortex (dmPFC), ventromedial prefrontal cortex (vmPFC), and an intermediate area (imPFC) spherical ROIs were grown to 8 mm based on peak voxels from previous studies for MVPA. Please note that small dmPFC and vmPFC spherical ROIs grown to 5 mm were used to extract GLM effect sizes. Anatomical ROIs for dmPFC and vmPFC were also taken from previous studies. COG stands for center of gravity. Coordinates were reported in accordance with the Montreal Neurological Institute (MNI) Atlas.
