# Supplementary material for: The dorsomedial prefrontal cortex computes task-invariant relative subjective value for self and other
Source: eLife. 2019 Jun 13;8:e44939. doi: 10.7554/eLife.44939 (PMC6565363; doi:10.7554/eLife.44939)
Supplement: Figure 4—source data 2. — Analysis types include Self and Other trials from the intertemporal or risky choice tasks. 8 mm spherical regions of interest (ROIs) include primary somatosensory cortex (S1), ventromedial prefrontal cortex (vmPFC), dorsomedial prefrontal cortex (dmPFC), and an intermediate ROI placed in the medial prefrontal cortex between the vmPFC and dmPFC (imPFC). [file elife-44939-fig4-data2.pdf]

Figure 4–source data 2: MVPA significance for online relative subjective value analyses

Analysis	ROI	Participant Mean	Null Mean	*P*
Intertemporal Self	S1	0.5177	0.5001	0.1906
	vmPFC	0.5228	0.5007	0.1385
	imPFC	0.5219	0.4996	0.1380
	dmPFC	0.5581	0.5000	0.0035
Intertemporal Other	S1	0.4956	0.4997	0.5753
	vmPFC	0.5181	0.4980	0.1649
	imPFC	0.5300	0.5003	0.0770
	dmPFC	0.5474	0.4994	0.0087
Risk Self	S1	0.5091	0.5001	0.3271
	vmPFC	0.5040	0.5010	0.4414
	imPFC	0.5357	0.4985	0.0307
	dmPFC	0.5472	0.4989	0.0070
Risk Other	S1	0.5067	0.4993	0.3395
	vmPFC	0.4905	0.4987	0.6542
	imPFC	0.5254	0.4993	0.0873
	dmPFC	0.5730	0.4984	0.0001

Related to Figure 4. Analysis types include Self and Other trials from the intertemporal or risky choice tasks. 8-mm spherical regions of interest (ROIs) include primary somatosensory cortex (S1), ventromedial prefrontal cortex (vmPFC), dorsomedial prefrontal cortex (dmPFC), and an intermediate ROI placed in the medial prefrontal cortex between the vmPFC and dmPFC (imPFC).
