# Supplementary material for: The dorsomedial prefrontal cortex computes task-invariant relative subjective value for self and other
Source: eLife. 2019 Jun 13;8:e44939. doi: 10.7554/eLife.44939 (PMC6565363; doi:10.7554/eLife.44939)
Supplement: Figure 5—source data 1. — Analysis types include training on Self and testing on Other trials, and vice versa, with the intertemporal and risky choice tasks. Also included are analyses training pattern classifiers on either intertemporal Self or Other trials and testing them on risk Self or Other trials, and vice versa. All pattern classifiers in this table were trained on data from the dorsomedial prefrontal cortex (dmPFC) 8 mm spherical region of interest. [file elife-44939-fig5-data1.pdf]

Figure 5–source data 1: MVPA significance for online relative subjective value analyses across agents and tasks

Analysis	Participant Mean	Null Mean	*P*
Intertemporal, train self test other	0.5590	0.4998	0.0021
Intertemporal, train other test self	0.5578	0.5010	0.0033
Risk, train self test other	0.5464	0.5006	0.0087
Risk, train other test self	0.5421	0.4982	0.0148
Train intertemporal self, test risk self	0.5633	0.5007	0.0196
Train intertemporal self, test risk other	0.5475	0.5003	0.0549
Train intertemporal other, test risk other	0.5558	0.5028	0.0450
Train intertemporal other, test risk self	0.5852	0.4988	0.0025
Train risk self, test intertemporal self	0.5688	0.5038	0.0207
Train risk self, test intertemporal other	0.5647	0.5009	0.0176
Train risk other, test intertemporal other	0.5577	0.4995	0.0316
Train risk other, test intertemporal self	0.5628	0.4997	0.0282

Related to Figure 5. Analysis types include training on Self and testing on Other trials, and vice versa, with the intertemporal and risky choice tasks. Also included are analyses training pattern classifiers on either intertemporal Self or Other trials and testing them on risk Self or Other trials, and vice versa. All pattern classifiers in this table were trained on data from the dorsomedial prefrontal cortex (dmPFC) 8-mm spherical region of interest.
